# Resistance exercise with different workloads have distinct effects on cellular respiration of peripheral blood mononuclear cells

**DOI:** 10.14814/phy2.15394

**Published:** 2022-07-19

**Authors:** Emilia Ilona Lähteenmäki, Max Koski, Iida Koskela, Elias Lehtonen, Anna Kankaanpää, Heikki Kainulainen, Simon Walker, Maarit Lehti

**Affiliations:** ^1^ Faculty of Sport and Health Sciences University of Jyväskylä Jyväskylä Finland; ^2^ Research Centre for Physical Activity and Health LIKES Jyväskylä Finland; ^3^ Biology of Physical Activity, Neuromuscular Research Center University of Jyväskylä Jyväskylä Finland; ^4^ Department of Biological and Environmental Science University of Jyväskylä Jyväskylä Finland; ^5^ Gerontology Research Center (GEREC), Faculty of Sport and Health Sciences University of Jyväskylä Jyväskylä Finland

**Keywords:** mitochondria, resistance training, white blood cells, bioenergetics, training volume

## Abstract

Little is known how acute exercise‐induced inflammation and metabolic stress affect immune cell bioenergetics and the portion of its components. Therefore, we investigated acute effects of eccentric‐only (E), concentric‐only (C) and combined eccentric‐concentric resistance exercise (E + C) bouts on cellular respiration of peripheral blood mononuclear cells (PBMCs). Twelve strength‐trained young men performed bench press resistance exercises in randomized order. Venous blood samples were drawn at pre‐, 5 min post‐ and 24 h post‐exercise. Several PBMC respiration states were measured using high‐resolution respirometry. Levels of leukocytes, interleukin 6 (IL‐6), C‐reactive protein (CRP), creatine kinase (CK), blood lactate and maximum voluntary isometric force were measured from the same time points. Effects of blood lactate and pH change on bioenergetics of PBMCs were investigated ex vivo. PBMC routine respiration (*p* = 0.017), free routine capacity (*p* = 0.025) and ET‐capacity (*p* = 0.038) decreased immediately after E + C. E responded in opposite manner 5 min post‐exercise compared to E + C (*p* = 0.013) and C (*p* = 0.032) in routine respiration, and to E + C in free routine activity (*p* = 0.013). E + C > C > E was observed for increased lactate levels and decreased isometric force that correlated with routine respiration (R = −0.369, *p* = 0.035; R = 0.352, *p* = 0.048). Lactate and pH change did not affect bioenergetics of PBMCs. Acute resistance exercise affected cellular respiration of PBMCs, with training volume and the amount of metabolic stress appear influential. Results suggest that acute inflammation response does not contribute to changes seen in cellular respiration, but the level of peripheral muscle fatigue and metabolic stress could be explaining factors.

## Graphical abstract

Resistance exercise with different workloads have distinct effect on cellular respiration of PBMCs. Training volume and amount of metabolic stress are influential.

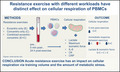

## 1 INTRODUCTION

In addition to increasing muscle strength and size, resistance training has several other health benefits such as reducing chronic low‐grade inflammation, which is associated with several metabolic diseases (Furman et al., 2019; Ihalainen et al., 2014). The anti‐inflammatory effect of resistance training is associated with tightly regulated interaction between muscle cells and leukocytes (Petersen & Pedersen, 2005; Pillon et al., 2013). A single bout of resistance exercise can trigger a local inflammation response, which stimulates the production of pro‐ and anti‐inflammatory cytokines (Freidenreich & Volek, 2012). Contracting muscle cells secrete interleukin‐6 (IL‐6), which activates the production of anti‐inflammatory cytokines IL‐1 receptor antagonist (IL‐1ra) and IL‐10 in immune cells that reduces the low‐grade inflammation state (Gleeson et al., 2011). Moreover, resistance exercise containing eccentric contractions (i.e., forced lengthening) are known to cause micro‐injuries in muscle cells, and the reparation and adaptation process also triggers large local inflammation response (Pillon et al., 2013; Willoughby & Taylor, 2004). When compared to concentric (shortening) contractions, more muscle damage occurs (Willoughby & Taylor, 2004), at least in those naïve to the exercise. Damage is associated with increased levels of serum markers, such as creatine kinase (CK) and inflammation marker C‐reactive protein (CRP) (Gould & Weiser, 2001; Willoughby & Taylor, 2004). Inflammation is followed by infiltration of immune cells to the damaged muscle area for removal of dead cell material (Pillon et al., 2013; Willoughby & Taylor, 2004). This is followed by an anti‐inflammatory phase, which is required for myogenesis and growth of myofibers leading to muscle regeneration (Chazaud, 2016; Pillon et al., 2013).

Peripheral blood mononuclear cells (PBMCs), consisting of lymphocytes, monocytes and dendritic cells, are an important component of the immune system and they have a central role in muscle repair and exercise related anti‐inflammatory response (Connolly et al., 2004). PBMCs respond to inflammation and stress by producing cytokines, chemokines and growth factors (Connolly et al., 2004). Indeed, both endurance and resistance exercise are known to elevate the number of PBMCs immediately after an exercise bout and strongly influence the gene expression related to pro‐ and anti‐inflammatory response, cell growth and tissue repair (Carlson et al., 2011; Connolly et al., 2004). Furthermore, PBMCs have been suggested to work as systemic biomarkers for metabolic stress, which can lead to changes in metabolic phenotype or even progression of the disease state (Chacko et al., 2013; Hill et al., 2019; Kramer et al., 2014). Underlying mechanisms causing changes in metabolism are not completely clear but it is known that mitochondria are sensitive to several metabolic and inflammatory stressors (Chacko et al., 2014) and these can have either a positive or negative influence on mitochondrial function. Exercise can be seen as a major stressor and its influence on bioenergetics has been studied widely among skeletal muscle but poorly in other cell types, such as PBMCs.

Information related to the effects of exercise on PBMCs' bioenergetics are limited and, thus far, unclear. Hedges et al. (2019) concluded that PBMCs are a poor marker of muscle mitochondrial function in humans when investigating effects and adaptation to high intensity interval training (Hedges et al., 2019) whereas the study of Tyrrell et al. (2016) showed that leukocytes' maximal respiration capacity correlated with skeletal and cardiac muscle bioenergetics (Tyrrell et al., 2016). However, it is clear that skeletal muscle cells and PBMCs have a strong connection, which is seen especially during and after exercise as leukocytosis, inflammation response and the muscle repair process (Gleeson et al., 2011; Pillon et al., 2013). These events could cause changes in bioenergetics of PBMCs. Moreover, the effects of peripheral muscle fatigue including depletion of ATP and metabolic acidosis (Finsterer, 2012) on bioenergetics of PBMCs has not been studied widely. It is known that exercise can temporarily elevate the number of circulating leukocytes (Finsterer, 2012) but the impact of biochemical factors related to fatigue on function of leukocytes is unclear.

To our knowledge, the effects of resistance exercise and different contraction types (i.e., eccentric and concentric) on cellular respiration has not been studied before. However, an exercise model comparing responses to eccentric and concentric exercise could reveal differences in bioenergetics of PBMCs accompanying the expected differences in metabolic cost and inflammation response. Consequently, our study investigated the acute effects of three resistance exercise bouts, eccentric‐only (E), concentric‐only (C) and combined eccentric and concentric contractions (E + C), on cellular respiration of PBMCs. It was hypothesized that metabolic cost and inflammation response to C versus E are different and would lead to different respiration responses of PBMCs. Combined eccentric and concentric contractions would reveal whether high metabolic cost and high inflammation response has an additive effect on cellular respiration of PBMCs. Finally, we studied ex vivo if lactate concentration and change in pH specifically affects cellular respiration of PBMCs.

## 2 METHODS

### 2.1 Participants

Twelve healthy male subjects were recruited to the study through local advertisement (age = 26.5 ± 3.6 year, height = 182.4 ± 6.3 cm, weight = 88.3 ± 10.0, muscle mass = 43.6 ± 5.3 kg, fat percentage = 13.8 ± 4.3% [InBody 770], maximum isometric force = 1084 ± 4.1 N). Inclusion criteria were experience in resistance training (>100 kg bench press or ≥1.25 times bodyweight). We hypothesized that inflammation response in untrained test subjects might vary largely, therefore only trained test subjects were chosen to obtain more homogenous responses. In addition, inclusion criteria included no use of anabolic agents such as steroids or medication known to affect the inflammatory status.

We estimated the appropriate *n* with calculated effect sizes for study design by using G*Power program (Faul et al., 2007). According to these calculations, the sample size of 12 would be sufficient to detect medium‐sized effect (Cohen *d =* 0.3) with 80% power.

All subjects gave their written informed consent prior to participation. All participants filled a PAR‐Q+ questionnaire (Goodman et al., 2011) to ensure their readiness for participation. The study was conducted according to the guidelines of the Declaration of Helsinki for research on human participants and was approved by the ethical committee of the University of Jyväskylä (9/2018). During measurements one participant was injured outside of the study and was not able to attend the last exercise testing. Therefore, for eccentric exercise *n* = 11.

### 2.2 Study design

**FIGURE 1:**
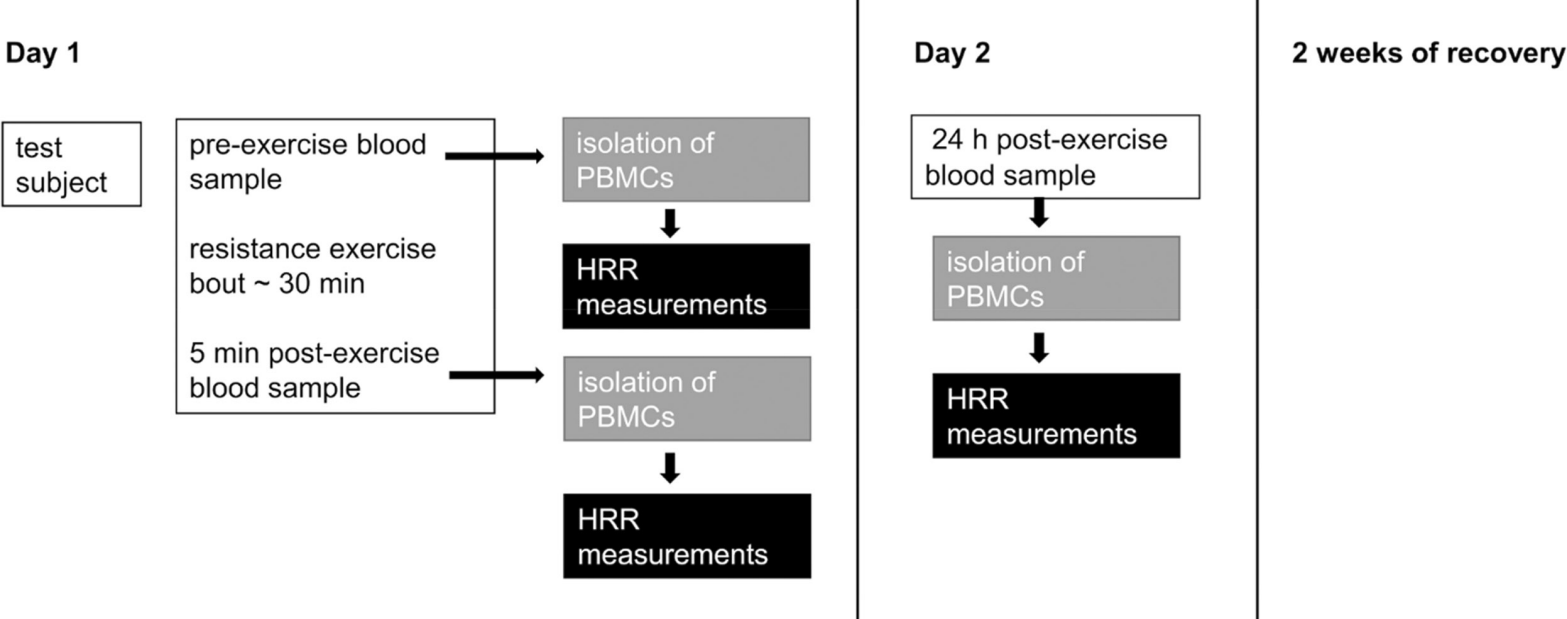
Timeline for study design. Figure represents timeline and study design for one test subject. Daily study design included resistance exercise bout, blood samples at two time points (pre‐ and 5 min post‐exercise), isolation of PBMCs and cellular respiration measurements (HRR high resolution respirometry). The next day included 24 h post‐exercise blood sample. After each resistance exercise bout, subjects had 2 weeks of recovery before next measurement days.

Before each exercise bout, subjects were instructed to enter the lab in a 12 h fasted state. Venous blood was drawn from an antecubital vein upon arrival for the pre‐exercise sample, and again 5 min and 24 h after each of the three exercise bouts (5 min post‐ and 24 h post‐exercise) (Figure 1). Tests of maximal isometric bench press force were taken pre‐, <1 min post‐, and 24 h post‐exercise. The assigned resistance exercise was isokinetic bench press with 5 sets of 10 maximum exertion repetitions. Subjects performed  (1) concentric‐only (C),   (2) eccentric‐only (E), or   (3) combined eccentric‐concentric (E + C) contraction exercise bouts in a randomized order. Each exercise bout was separated by 14 days to ensure no residual effects from the previous bouts (Figure 1). To ensure standardized nutrition prior to each exercise bouts, subjects completed a 24 h food diary prior to the first exercise bout and were instructed to repeat the exact same meal plan (the diary was photocopied and returned to the subjects) prior for the following two exercise bouts. Subjects were asked to avoid any other resistance training activities targeting the pectoral and triceps muscles during the period of study.

### 2.3 Exercise protocol

Before performing the exercise protocols, a two‐minute warm‐up was performed using submaximal contractions on the custom‐made isokinetic smith‐machine (Faculty of Sport and Health Sciences, University of Jyväskylä). All exercise protocols were completed with this isokinetic bench press device. The machine operated with a speed of 0.1 m/s in all three exercises, independent of the force produced against it. Ground reaction forces were recorded with a 2‐D strain gauge force plate (Faculty of Sport and Health Sciences, University of Jyväskylä) positioned under the bench. During the concentric and eccentric loadings, the bar's movement paused for 500 ms at the top and bottom position, to aid pacing the contractions. In E, subjects were asked to start to maximally contract their muscles, once the bar stopped at the top position and continue pushing as hard as possible until the bar stopped at the bottom position (i.e., approx. 0.5 s isometric contraction prior to eccentric). In C, subjects were asked to maximally contract their muscle once the bar stopped at the bottom position and continue pushing until the bar stopped at the up position. During the unloaded traveling phase of the machine, subjects were instructed to rest their arms while grasping the bar to avoid activation of the targeted muscles. During E + C, only 1 ms ‘*pauses’* were utilized at the top and bottom position to ensure constant bar movement throughout the set. Here, subjects were instructed to push with maximal effort continuously throughout each set. All three exercise protocols consisted of five sets of ten maximal repetitions with 2 min inter‐set rest. During rest periods, subjects remained supine on the bench with minimal arm movement.

### 2.4 Maximal isometric force

Maximal isometric force was recorded at 90° of elbow flexion, measured with a custom‐made isometric bench press (Hulmi et al., 2017), equipped with two strain gauge force transducers on both sides (Faculty of Sport and Health Sciences, University of Jyväskylä) before exercise (pre), 1 min (1 min post‐) and 24 h (24 h post‐) after exercise. The subjects performed three maximal isometric contractions lasting from 3 to 5 s, with 15 s inter‐trial rest. The highest force value from the trials was recorded as maximum voluntary contraction. The isometric maximal repetitions were performed on a bench press with feet on the bench and the back flat. Force signals were collected via an analog‐to‐digital converter (Micro 1401, Cambridge Electronic Design Ltd) into Signal software (version 4.10, Cambridge Electronic Design Ltd) and were sampled at 1000 Hz. Analyses were performed offline using a customized script and pre‐filtering with a 10 Hz 4th‐order low‐pass Butterworth filter.

### 2.5 Blood collection and blood parameters

Blood samples were collected for the isolation of PBMCs and for detection of IL‐6, CK and CRP levels. Blood samples (30 ml) were drawn from the antecubital vein from each subject while seated pre‐, 5 min post‐ and 24 h post‐exercise to Vacutainer VACUETTE K3EDTA (Greiner Bio‐One) tubes containing anticoagulant (EDTA). Whole blood count and the number of lymphocytes and monocytes were assessed by Sysmex XP‐300 (Sysmex Corporation) to obtain the number of leukocytes and platelets. 18 ml of blood was used for isolation of PBMCs and 12 ml for serum isolation.

For the detection of IL‐6, CK and CRP, serum was isolated from blood samples by centrifugation (10 min 2200 *g*). Serum was stored in −20°C for later analysis, which was performed using IMMULITE 2000 XPi Immunoassay System (Siemens Healthcare GmbH) according to manufacturers´ instructions. Lactate was measured from fingertip blood samples, which were collected into capillary tubes (20 μl) at all three time points. Samples were then placed in a 1 ml hemolyzing solution and analyzed automatically with Biosen C‐line lactate analyzer (EKF diagnostic, Biosen).

### 2.6 Isolation of PBMCs

Isolation of PBMCs was performed in two sets, where both isolation sets included three samples. Prior to handling, blood samples stood in containers at room temperature protected from light. Eighty‐two minutes was the longest standing time for blood samples due to the daily measurement schedule. The effect of standing time on cellular respiration of PBMCs was tested with paired samples T‐test (SPSS, version 24 for Microsoft Windows, IBM). Standing time did not have a statistical effect on cellular respiration.

PBMCs were isolated in 50 ml Leucosep tubes (Greiner Bio‐One) with 15 ml of Ficoll‐Paque TM PLUS (GE Healthcare) (Sumbalova et al., 2020). Eighteen ml of blood diluted in the same amount RPMI‐1640 medium (Sigma Aldrich) was centrifuged at 1000 *g* (10 min, acceleration 6, no brakes). The layer of PBMCs and platelets was collected and washed twice with RPMI‐1640 (120 *g*, 10 min, acceleration 9, brake 6) in order to reduce PLT/PBMC ratio below 7 (Sumbalova et al., 2020). PBMC‐platelet pellet was resuspended in 0.5 ml RPMI‐1640 and the amount of PBMCs and platelets was obtained with Sysmex XP‐300 (Sysmex Corporation). Viability of PBMCs was tested using the Trypan blue (Thermo Fisher Scientific) and average viability was 96.5%. Cell suspension was diluted in RPMI‐1640 so that the cell concentration was 2 × 10^6^/ml.

### 2.7 Cellular respiration of PBMCs

The cellular respiration of intact PBMCs was measured using a high‐resolution respirometer (HRR, O2k FluoRespirometer, Oroboros Instruments). Substrate‐uncoupler‐inhibitor titration 3 (SUIT3) protocol designed for intact cells was chosen for measurements (Doerrier & Gnaiger, 2014). The data were collected with the application of DatLab software version 7.3.0.3 (Oroboros Instruments). For the characterization of cellular respiration, several respiration states were analyzed. Cell suspension was added to chambers and let to balance for routine respiration (R). Oligomycin (0.015 μM, Sigma Aldrich) was added in order to inhibit ATP synthase and induce leak respiration (L). This illustrates proton leak, which is not used for ATP production. With titration of optimum concentrations of the uncoupler carbonyl cyanide m‐chlorophenyl hydratzone (CCCP) (0.25–2.0 μM, Sigma Aldrich), maximum electron transfer capacity was induced (ET). Finally, rotenone (0.5 μM, Sigma Aldrich) and antimycin A (2.5 μM, Sigma Aldrich)‐ were added to inhibit the function of respiration complexes I and III. This was applied to determine residual oxygen consumption (ROX), which is respiration due to oxidative side reactions remaining after inhibition of the electron transfer pathway and it was subtracted from respiration states (Doerrier & Gnaiger, 2014). All measurements were performed at 37°C in RPMI‐1640 medium. Absolute respiration values were normalized for the cell amount per chamber, which was counted with Sysmex XP‐300 (Sysmex Corporation).

Free routine activity $\left(R-L\right)$ and excess ET‐R capacity $\;\left(\mathrm{ET}-R\right)\;$ were calculated via the above‐ mentioned states. Following flux control ratios were calculated for an evaluation of respiratory parameters independent of cell size and mitochondrial content (Gnaiger, 2020). These were R/ET control ratio, L/R coupling control ratio and L/ET coupling control ratio. We also calculated following flux control efficiencies: ET‐R control efficiency ([ET‐R]/ET) and R‐L control efficiency ([R‐L]/R) (Gnaiger, 2020).

### 2.8 Effect of lactate and pH change on the cellular respiration of PBMCs

Additionally, it was of interest to study whether accumulation of lactate or change of blood pH could have been a trigger for changes observed in the cellular respiration of PBMCs. Here, we collected resting fasted blood samples (18 ml) from five resistance trained men and isolated PBMCs and measured the cellular respiration by using the same methods as described above.

To test whether lactate had an impact on the cellular respiration of PBMCs, 20 mmol of sodium L‐lactate (Sigma Aldrich) was added to the HRR measurement chamber after measurements of routine respiration of PBMCs and SUIT3 protocol was completed (Doerrier & Gnaiger, 2014). The concentration of sodium L‐lactate was determined based on the probable physiological level of lactate after vigorous exercise (Goodwin et al., 2007).

The effect of pH change on the cellular respiration of PBMCs was assessed by adding 10 mmol of L‐(+)‐lactic acid (Sigma Aldrich) to the HRR measurement chamber after routine respiration of PBMCs and SUIT3 protocol was completed. According to studies, a vigorous exercise can drop the pH of blood near 7.10 and could be under 7.0 in skeletal muscle (Robergs et al., 2005). The concentration of lactic acid was chosen based on measurement with RPMI‐1640 medium where L‐(+)‐ lactic acid was titrated until the pH of the medium (7.91) dropped to 7.01. pH was measured with Mettler Toledo FiveEasy Plus (Mettler Toledo). The same respiration states were analyzed in both experiments.

### 2.9 Statistical analyses

Statistical analyses were carried out using the Statistical Package for Social Sciences (SPSS version 24 and 28 for Microsoft Windows, IBM). Values are reported as mean ± standard deviation (SD), unless otherwise stated. All values were verified for outliers, which were excluded from the analysis (>3× interquartile range [IQR]). Significance level was set at 0.05 in all analyses. ANOVA with repeated measures was used to examine whether there were differences in the study variables between the groups over time (group [exercises E, C and E + C] by time [repeated factor: pre, 5 min post and 24 h post]). Mauchly's test was used to test the assumption of sphericity and Greenhouse–Geisser corrections were applied if the assumption was violated. To study the effect of time separately within resistance exercises and group × time interaction between adjacent time points, within‐subject contrasts were used as post hoc comparison. Hedges' *g* utilizing Hedge's correction factor recommended for small sample sizes was calculated for significant differences to assess the effect size. The effect size of 0.2 is considered small, 0.5 medium and 0.8 large. Pearson's correlation coefficient was used to determine relationships between respiration states and lactate, isometric force, CK and CRP. Independent‐Samples T‐test and Paired‐Samples T‐test were used to study if lactate and pH change caused by lactic acid had an effect on the cellular respiration of PBMCs. All graphs were generated using GraphPad Prism 9.1.0 (GraphPad Software).

## 3 RESULTS

### 3.1 Cellular respiration of PBMCs

Cellular respiration of PBMCs was analyzed through several respiration states (Table 1), which illustrate the function of electron transfer pathway states. Respiration states were analyzed between time points (pre‐, 5 min post‐ and 24 h post‐exercise) and resistance exercises (E, C and E + C). Table 1 shows main effects of group and time, and group × time interaction on each respiration state.

**TABLE 1:** Main effects of group and time, and their interaction on respiration states

	Group	Time	Interaction
Routine respiration	F(_2_) = 5.3, *p* = 0.015	F(_2_) = 1.1, *p* = 0.339	F(_4_) = 4.2, *p* = 0.007
Leak respiration	F(_2_) = 2.9, *p* = 0.081	F(_2_) = 0.9, *p* = 0.434	F(_4_) = 0.6, *p* = 0.644
Free routine activity	F(_2_) = 1.6, *p* = 0.232	F(_2_) = 0.9, *p* = 0.412	F(_4_) = 1.4, *p* = 0.266
Excess ET‐R capacity	F(_2_) = 0.0, *p* = 0.998	F(_2_) = 1.3, *p* = 0.294	F(_4_) = 0.9, *p* = 0.462
ET‐capacity	F(_2_) = 0.4, *p* = 0.686	F(_2_) = 5.8, *p* = 0.015	F(_4_) = 1.8, *p* = 0.228

*Note*: *n =* 10.

**FIGURE 2:**
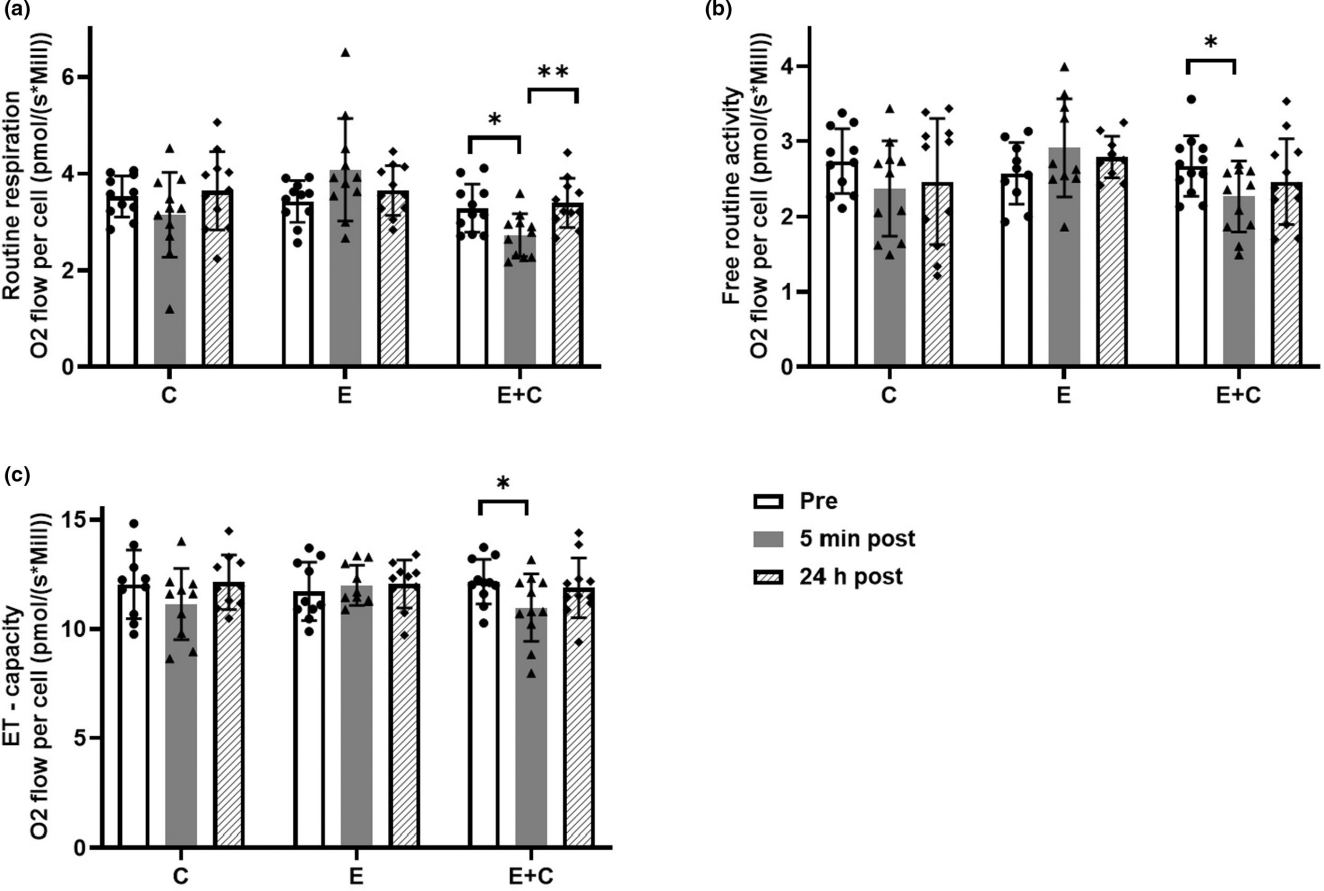
Respiration states of PBMCs at different time points of the studied resistance exercises. Graphs represent (a) routine respiration (*n* = 11), (b) free routine activity (*n* = 10–12) and (c) ET‐capacity (*n* = 10–11) in different resistance exercises (E eccentric, C concentric and E + C combination). Different colored bars indicate time points (pre‐, 5 min post‐ and 24 h post‐exercise) along with individual subject data. Data are means ± SD. *p < 0.05 and **p < 0.01 between line marked groups; repeated measures ANOVA with main effect of time.

Post hoc analysis showed that routine respiration (F(_1_) = 8.2, *p* = 0.017, *g =* −0.83), free routine activity (F(_1_) = 6.8, *p* = 0.025, *g =* −0.73) and ET‐ capacity (F(_1_) = 5.7, *p* = 0.038, *g =* −0.69) decreased significantly immediately after E + C (Figure 2). C and E responses did not reach statistical significance. Routine respiration had also significant difference in E + C between 5‐min post‐ and 24 h post‐exercise (F(_1_) = 10.8, *p* = 0.008, *g =* 0.95) (Figure 2a). There were no significant changes in other respiration states between time points.

**FIGURE 3:**
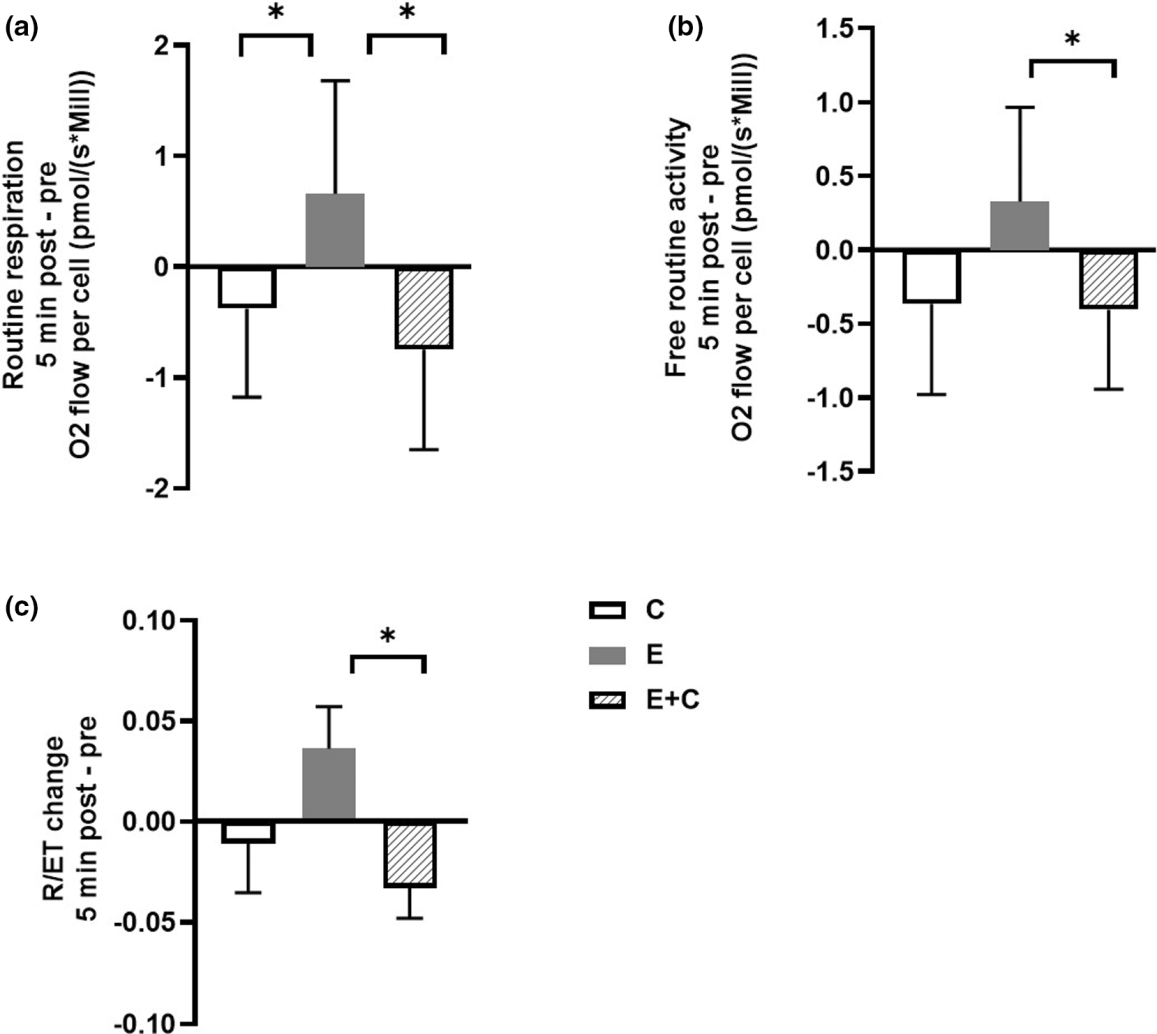
Change in respiration states and routine control ratio immediately after resistance exercises. Figure shows changes (5 min post—pre) in (a) routine respiration (*n* = 10), (b) free routine capacity (*n* = 10) and (c) routine control ratio (*n* = 9) after resistance exercises. Different colored bars indicate exercises (E eccentric, C concentric and E + C combination). Data are means ± SD. *p < 0.05 between line marked groups; repeated measures ANOVA with interaction (group × time).

Changes (∆) in respiration states immediately after resistance exercise (from pre‐exercise to 5 min post‐exercise response) were compared between resistance exercises (Figure 3). Post hoc comparison indicated that E was significantly different when compared to C (F(_1_) = 6.5, *p* = 0.032, *g =* 0.73) and E + C (F(_1_) = 9.6, *p* = 0.013, *g =* 0.94) in routine respiration (Figure 3a). Free routine activity was also significantly different between E and E + C (F(_1_) = 9.5, *p* = 0.013, *g =* 0.87) (Figure 3b).

Several flux control ratios were analyzed between time points and resistance exercises. Flux control ratios illustrate the ratio of oxygen flux normalized to the maximum flux and are used as normalization independent of mitochondrial content. Change in routine control ratio showed significant difference between E and E + C (F(_1_) = 7.4, *p* = 0.026, *g =* 1.17) (Figure 3c) when 5 min post‐exercise response was compared between resistance exercises. There were no significant changes in other flux control ratios.

### 3.2 Inflammatory and muscle injury markers, maximum isometric force and blood lactate

CRP, CK and IL‐6 levels, maximum isometric force and blood lactate levels were analyzed between time points and resistance exercises. Table 2 shows main effects of group and time, and their interaction on these variables.

**TABLE 2:** Main effects of group and time, and their interaction on inflammatory and muscle injury markers, maximum isometric force and blood lactate

	Group	Time	Interaction
CRP	F(_1.185_) = 0.4, *p* = 0.582^a^	F(_1.018_) = 1.2, *p* = 0.312^a^	F(_1.087_) = 1.6, *p* = 0.240^a^
CK	F(_2_) = 0.4, *p* = 0.660	F(_1.080_) = 3.5, *p* = 0.100^a^	F(_1.603_) = 0.2, *p* = 0.765^a^
IL‐6	F(_2_) = 1.5, *p* = 0.245	F(_2_) = 0.5, *p* = 0.639	F(_4_) = 0.8, *p* = 0.557
Maximum isometric force	F(_2_) = 12.2, *p* < 0.001	F(_1.201_) = 137.2, *p* < 0.001^a^	F(_4_) = 18.6, *p* < 0.001
Blood lactate	F(_2_) = 10.4, *p* = 0.001	F(_1.144_) = 176.6, *p* < 0.001^a^	F(_2.040_) = 9.1, *p* = 0.002^a^

^a^ Greenhouse–Geisser corrected, *n =* 10.

#### 3.2.1 Inflammatory and muscle injury markers

Main effects of group and time, and their interaction did not show statistical significances on CRP, CK or IL‐6, hence there were no major changes between time points or between resistance exercises. Mean values and SDs of the data are represented in supplementary data (Publicly available DOI for Figshare data: https://doi.org/10.6084/m9.figshare.16586411.v1).

#### 3.2.2 Maximum isometric force

**FIGURE 4:**
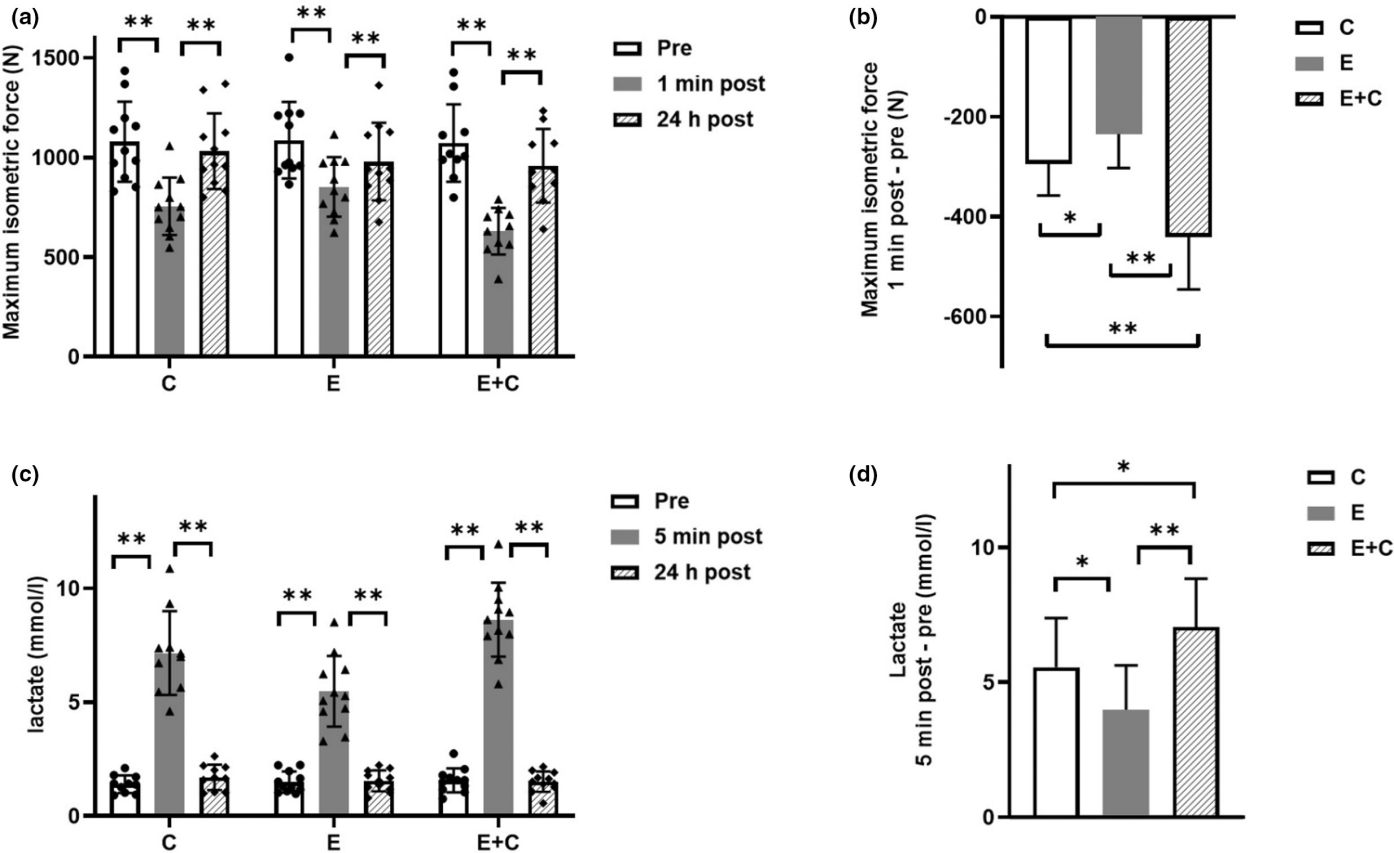
Isometric force, blood lactate and their change after resistance exercises. Figure represents (a) isometric force at each time point in three resistance exercises along with individual subject data (*n =* 10–11), (b) change in isometric force after resistance exercises (1 min post—pre) (*n =* 10), (c) lactate levels in each time point in three resistance exercises along with individual subject data (*n =* 10–11) and (d) change in lactate levels after resistance exercises (5 min post—pre) (*n =* 10). Different colored bars indicate time points (pre‐, 1/5 min post‐ and 24 h post‐ exercise) or resistance exercises (E eccentric, C concentric and E + C combination). Data are means ± SD. *p < 0.05 and **p < 0.01; repeated measures ANOVA with main effect of time and interaction (group × time).

According to main effects and interaction (Table 2), maximum isometric force was different between the groups. Post hoc analysis showed that isometric force decreased significantly immediately after all resistance exercises (C: −27 ± 6%, F(_1_) = 89.3, *p* < 0.001, *g =* −2.74; E: −22 ± 6%, F(_1_) = 131.2, *p* < 0.001, *g =* −3.32; E + C: −41 ± 10%, F(_1_) = 160.6, *p* < 0.001, *g =* −3.84) and this was reversed at 24 h post‐exercise (*p* < 0.001, Figure 4a).

Changes (∆) in isometric force (from pre‐ to 5 min post‐exercise response) were compared between resistance exercises (Figure 4b). E + C (F(_1_) = 46.1, *p* < 0.001, *g =* −2.14) and C (F(_1_) = 6.4, *p* = 0.032, *g =* −0.79) showed a statistically greater decrease than E (Figure 4b). Also, the decrease from C was less than the decrease from E + C (F(_1_) = 13.8, *p* = 0.005, *g =* 1.19) (Figure 4b).

#### 3.2.3 Blood lactate

According to main effects and interaction (Table 2), blood lactate levels were different between the groups. Post hoc comparisons showed that lactate levels increased significantly immediately after all resistance exercises (C: F(_1_) = 103.1, *p* < 0.001, *g =* 3.08; E: F(_1_) = 64.0, *p* < 0.001, *g =* 2.32; E + C: F(_1_) = 171.6, *p* < 0.001, *g =* 3.80) (Figure 4c) and this was reversed at 24 h post‐exercise (*p* < 0.001, Figure 4c).

Changes (∆) in lactate levels (from pre‐ to 5 min post‐exercise response) were compared between resistance exercises (Figure 4d). E + C (F(_1_) = 14.8, *p* = 0.004, *g =* 1.27) and C (F(_1_) = 5.3, *p* = 0.047, *g =* 0.70) showed a statistically greater increase than E. Also, the increase from E + C was greater than the increase from C (F(_1_) = 5.2, *p* = 0.048, *g =* 0.69) (Figure 4d).

### 3.3 Pearson's product moment correlation

Pearson correlation coefficients for 5 min post‐exercise response in routine respiration, free routine activity and ET‐capacity in relation to 5 min post‐exercise response in lactate, isometric force and inflammation and muscle injury markers are presented in Table 3. There was a significant negative correlation between lactate and routine respiration and a significant positive correlation between isometric force and routine respiration (Table 3).

**TABLE 3:** Pearson correlation coefficients for 5 min post‐exercise response (5 min post—pre) in respiration states in relation to 5 min post‐exercise response (5 min post—pre) in lactate, isometric force and inflammation and muscle injury markers

	Routine respiration (5 min post—pre)		Free routine activity (5 min post—pre)		ET‐ capacity (5 min post—pre)	
(5 min post—pre)	R	*p*	R	*p*	R	*p*
Blood lactate (mmol/L)	−0.369	0.035	−0.204	0.254	−0.163	0.365
Maximum isometric force (N)	0.352	0.048	0.258	0.154	0.205	0.260
CRP (mg/L)	−0.069	0.702	0.092	0.610	−0.231	0.195
CK (u/L)	0.093	0.608	0.011	0.953	0.256	0.150
IL‐6 (pg/ml)	0.018	0.919	−0.119	0.510	0.108	0.550

Abbreviations: *p*, p‐value; R, correlation coefficient.

### 3.4 Effect of lactate and pH change on the cellular respiration of PBMCs

**FIGURE 5:**
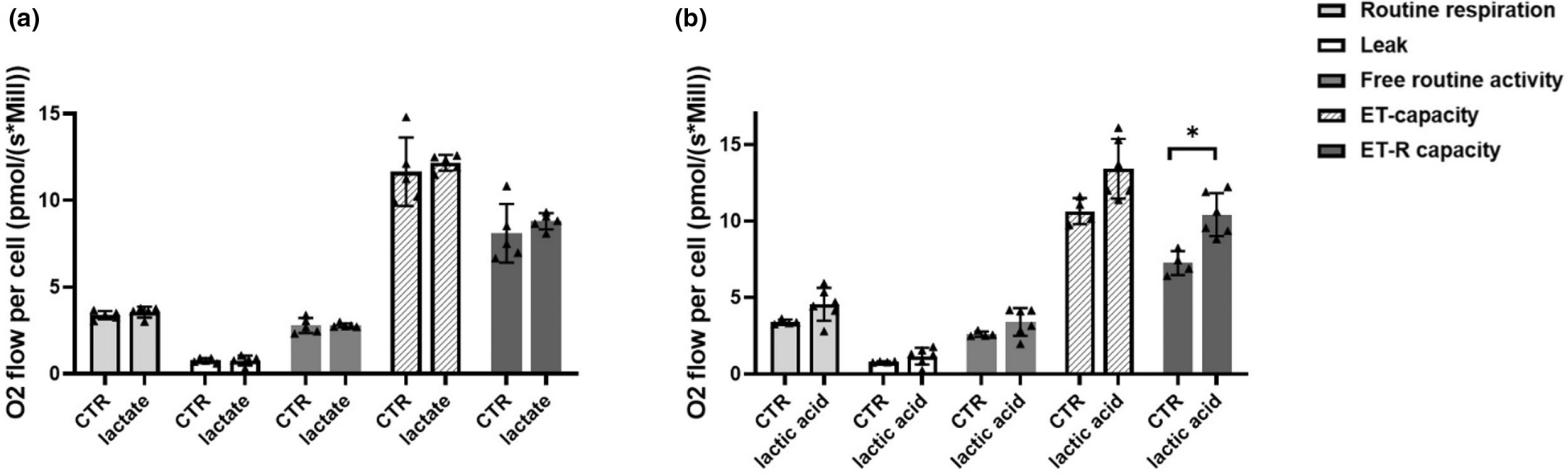
Effects of lactate and pH change on the cellular respiration states of PBMCs. Figure represents the effects of (a) lactate (*n* = 5) and (b) pH decrease (*n* = 4–6) on the cellular respiration states. Different shaded bars indicate respiration states (routine respiration, leak, free routine activity, ET—capacity and ET‐R capacity). Data are means ± SD. *p < 0.05; independent‐samples T‐test and paired‐samples T‐test.

Respiration states of lactate treated and non‐treated PBMCs were compared to study if changes on the cellular respiration of PBMCs that were observed after resistance exercises were mediated by lactate. The effect of pH change on the cellular respiration of PBMCs was also studied by comparing lactic acid treated samples to non‐treated samples. Lactate did not have a significant effect on respiration states (Figure 5a). The effect of pH change on the cellular respiration of PBMCs was also studied by comparing lactic acid treated samples to non‐treated samples. The decrease in pH caused by L‐lactic acid significantly increased ET‐R capacity only (t(_3_) = −3.583, *p* = 0.035) (Figure 5b).

### 3.5 Whole blood white blood cell, lymphocyte and monocyte counts

WBCs, as well as lymphocytes and monocytes, which are the main components of PBMCs, were counted at each time point and analyzed between timepoints and resistance exercises. The effect of plasma volume change was examined (according to Dill & Costill [1974] equation) (Dill & Costill, 1974; Matomäki et al., 2018) but this did not change the results, hence uncorrected data are presented. Table 4 shows main effects of group and time, and group × time interaction on these cell groups.

**TABLE 4:** Main effects of group and time, and their interaction on WBCs, lymphocytes and monocytes

	Group	Time	Interaction
WBCs	F(1.214) = 1.4, *p* = 0.275^a^	F(_2_) = 18.3, *p* < 0.001	F(_4_) = 4.2, *p* = 0.006
Lymphocytes	F(1.170) = 0.6, *p* = 0.498^a^	F(1.317) = 15.3, *p* = 0.001 ^a^	F(_4_) = 5.7, *p* = 0.001
Monocytes	F(1.197) = 0.2, *p* = 0.746^a^	F(_2_) = 5.1, *p* = 0.016	F(1.872) = 0.3, *p* = 0.763^a^

^a^ Greenhouse–Geisser corrected *n =* 11.

According to post hoc tests, the number of WBCs increased after each resistance exercise (C: F(_1_) = 37.4, *p* < 0.001, *g =* 1.70; E: F(_1_) = 9.9, *p* = 0.010, *g =* 0.91; E + C: F(_1_) = 18.7, *p* = 0.001, *g =* 1.20) (Table 5). The difference between 5 min post‐ and 24 h post‐exercise reached statistical significance in E (F(_1_) = 5.7, *p* = 0.039, *g =* −0.72) and E + C (F(_1_) = 9.0, *p* = 0.012, *g =* −0.84). In C, the difference between pre‐ and 24 h post‐exercise WBC counts were also significantly different (F(_1_) = 14.0, *p* = 0.003, *g =* 1.04) (Table 5).

**TABLE 5:** WBCs, lymphocyte and monocyte counts in pre‐, 5 min post‐ and 24 h post‐exercise

Leukocytes (10^6^ cells/ml)	Pre mean (SD)	Post‐5 min mean (SD)	Post 24 h mean (SD)	Change (∆) between exercises
WBCs				
C	4.9 ± 1.1*	5.6 ± 1.2	5.4 ± 1.3^$^	E ‐ E+C*
E	5.0 ± 1.1*	5.6 ± 1.2^#^	5.1 ± 1.1	C ‐ E+C*
E + C	5.4 ± 1.1*	6.6 ± 1.6^#^	5.6 ± 1.2	
Lymphocytes				
C	1.9 ± 0.5*	2.2 ± 0.6	2.2 ± 0.5^$^	C ‐ E+C*
E	1.9 ± 0.6*	2.3 ± 0.8	2.0 ± 0.5	
E + C	2.1 ± 0.5*	2.8 ± 0.8^#^	2.1 ± 0.6	
Monocytes				
C	0.5 ± 0.2*	0.6 ± 0.2	0.6 ± 0.2	
E	0.5 ± 0.2*	0.6 ± 0.2	0.6 ± 0.2^$^	
E + C	0.6 ± 0.2	0.7 ± 0.2	0.6 ± 0.2	
Percentage in relation to WBCs amount				
Lymphocytes				
C	38	39	39	
E	38	40	40	
E + C	38	41	37	
Monocytes				
C	11	11	12	
E	10	11	12	
E + C	11	10	11	

*Note*: Significant differences (*p* < 0.05) between timepoints are shown in following symbols: *pre versus post‐5 min, ^#^post‐5 min versus post 24 h and $ pre versus post 24 h. Exercises are marked E = eccentric, C = concentric and E + C = combination. *n* = 11.

Each resistance exercise elevated the number of lymphocytes (C: F(_1_) = 18.6, *p* = 0.001, *g =* 1.16; E: F(_1_) = 8.8, *p* = 0.014, *g =* 0.83; E + C: F(_1_) = 20.6, *p* = 0.001, *g =* 1.22) (Table 5). The difference between 5 min post‐ and 24 h post‐exercise reached statistical significance in E + C only (F(_1_) = 11.7, *p* = 0.006, *g =* −0.92). The difference between pre‐ and 24 h post‐exercise lymphocyte count was significantly different in C (F(_1_) = 11.1, *p* = 0.007, *g =* 0.89). The number of monocytes was elevated after C (F(_1_) = 6.5, *p* = 0.027, *g =* 0.68) and E (F(_1_) = 5.3, *p* = 0.045, *g =* 0.64) and pre‐ and 24 h post‐exercise count reached statistically significant difference in E (F(_1_) = 7.7, *p* = 0.019, *g =* 0.77).

Change (∆) in WBC, lymphocyte and monocyte counts (5 min post‐ exercise response) were compared between resistance exercises (Table 5). Post hoc analyses showed that the number of WBCs was significantly higher after E + C when compared to C (F(_1_) = 11.7, *p* = 0.007, *g* = 0.66) and E (F(_1_) = 5.0, *p* = 0.048, *g* = 0.55). The number of lymphocytes was significantly higher after E + C when compared to C (F(_1_) = 10.1, *p* = 0.010, *g* = 0.66). There were no significant differences in the response between exercises among monocytes.

## 4 DISCUSSION

The purpose of this study was to investigate the effects of resistance exercise consisting of different contractions on cellular respiration of PBMCs, which is the first of its kind to our knowledge. The results show that combined eccentric‐concentric (E + C) contractions caused an acute decrease in cellular respiration of PBMCs as assessed by routine respiration, free routine capacity and ET‐capacity (Figure 2). Results also indicate that different contractions lead to different acute responses in cellular respiration of PBMCs. Overall, E + C and concentric‐only (C) exercise demonstrated acute decreases in respiration, whereas eccentric‐only (E) resistance exercise led to small (n.s.) increases in routine respiration and free routine capacity. These divergent acute responses being significant between‐groups 5 min post‐exercise (Figure 2).

Changes seen in routine control ratio were comparable with respiration results normalized to cell concentration (Figure 3c). E + C caused statistically significant lower routine control ratio in relation to E, which is a result of lower routine respiration values when normalized to cell amount (Figure 3c). Together, the results suggest that E + C caused decreased production of ATP via oxidative phosphorylation in PBMCs in relation to E, which is the reason why routine respiration was not operating as closely to ET‐capacity as in E (Gnaiger, 2020).

### 4.1 Inflammation response and cellular respiration of PBMCs

PBMCs have a central role in muscle repair and exercise‐related anti‐inflammatory response by producing anti‐inflammatory cytokines (Carlson et al., 2011; Connolly et al., 2004). It is also known that eccentric contractions cause more muscle micro‐damage than concentric contractions; triggering a stronger inflammation response (Franchi et al., 2017; Willoughby & Taylor, 2004). To study inflammation as a proxy for the amount of muscle micro‐injuries, and if these correlated with cellular respiration results, we compared the levels of IL‐6, CK and CRP (Table 2) at all three timepoints and between exercises. IL‐6 is secreted heavily from contracting muscle cells and is a key factor causing the anti‐inflammatory effect of exercising by activating anti‐inflammatory cytokine production in immune cells (Gleeson et al., 2013). IL‐6 has a specific receptor, IL‐6R, which is expressed in leukocytes and consequently can affect the function of these cells (Gleeson et al., 2013). Intensity and duration of the exercise are main factors affecting to IL‐6 response (Fischer, 2006). However, duration is the most dominant factor increasing IL‐6 concentration (Fischer, 2006), which could partly explain why there were no significant changes in IL‐6 levels after resistance exercises. Although the intensity of resistance exercises in our study was high, the duration was 30 min, which may be too short to induce an IL‐6 response in already trained subjects.

CK is one of the markers of muscle damage and CRP is an important biomarker of inflammation, activated by tissue injury (Baird et al., 2012; Haider et al., 2006). There were no significant responses in CK and CRP levels after resistance exercises indicating only minor inflammation and potentially little to no muscle damage (Table 2). Moreover, there were no significant differences between resistance exercises among these markers. We hypothesized that inflammation response could have caused a higher need for ATP in PBMCs especially after eccentric contractions because of increased production of cytokines. As there were no significant differences in 5 min and 24 h post‐exercise response between resistance exercises in any of these markers, and they did not correlate with respiration states (Table 3), this indicates that the (lack of) inflammation response does not explain the different cellular respiration of PBMCs after resistance exercises in trained subjects.

### 4.2 The role of skeletal muscle fatigue

We also measured the change in isometric force and blood lactate (Figure 4). Blood lactate is one of the biomarkers of peripheral muscle fatigue, which is described as a reversible muscle force loss during exercise (Finsterer, 2012). All resistance exercises caused significant elevation in blood lactate levels and significant decrease in maximum isometric force levels (Figure 4). As expected, E + C was the most fatiguing among all three exercises having almost twice the work of C and E. E + C caused the greatest elevation in lactate levels and the greatest drop in isometric force levels (−41 ± 10%), while C led to the second greatest elevation in lactate levels and drop in isometric force (−27 ± 6%). Completing the stepwise acute response between resistance exercises, E caused the lowest increase in lactate levels and the smallest decrease in isometric force (−22 ± 6%). Concentric contractions have been shown to be more energy‐consuming and, thus, metabolically demanding than eccentric ones (Franchi et al., 2017), which is reflected in our acute blood lactate and maximum muscle force results between C and E (Franchi et al., 2017; Souron et al., 2018). Furthermore, the difference between resistance exercises can be seen in cellular respiration results (Figures 2 and 3). Even though only E + C significantly reduced cellular respiration, C had a similar effect as seen by the trend. Results suggest that the effect of C was more determinative than the effect of E in cellular respiration of PBMCs.

On the whole, the observed acute blood lactate responses and reductions in maximum isometric force were as predicted prior to the study. While the lactate levels observed 5 min post‐exercise (5–9 mmoL/L) were somewhat lower than a previous study (10–12 mmoL/L) (Walker et al., 2013), it should be remembered that the upper body muscles are smaller than those of the lower body. Secondly, the observed ~41% decrease in maximum force in the present study is similar to that observed following 40 maximum isometric bench press contractions in Häkkinen et al. (1998) and reflective of the large fatiguability of trained subjects (compared to untrained subjects) (Ahtiainen et al., 2011; Häkkinen et al., 1998).

The change in blood lactate (from pre‐exercise to 5 min post‐exercise response) correlated negatively with the change in routine respiration (Table 3), indicating that higher metabolic stress is accompanied by lower respiration of PBMCs. Further, the change in isometric force correlated positively with the change in routine respiration (Table 3), showing that greater muscle fatigue is also associated with lower routine respiration levels. These findings suggest that the severity of the resistance exercise influences the bioenergetics of PBMCs and may partly explain the differences in the response between contractions; rather than the contraction modes per se. Interestingly, Sakharov et al. (2012) studied how endurance training above and below the anaerobic threshold affects gene expression levels of white blood cells in athletes (Sakharov et al., 2012). They found that the training protocol leading to exhaustion upregulated more genes related to stress and inflammation than the moderate protocol (Sakharov et al., 2012). In addition, they concluded that training leading to exhaustion caused greater changes in blood lactate, and serum growth hormone and cortisol levels. Hence, the amount of muscle fatigue and metabolic challenge of the exercise could influence cellular respiration of PBMCs.

### 4.3 Lactate and change in pH

Peripheral muscle fatigue is a result of several mechanisms including metabolic acidosis and reduction of ATP (Finsterer, 2012). When the need for ATP exceeds the capacity of muscle mitochondria to synthesize it via oxidative phosphorylation, ATP is generated from anaerobic glycolysis and the phosphagen system (Robergs et al., 2004). Hydrolysis of ATP from these non‐mitochondrial sources leads to proton accumulation. Together with disturbances in the balance of proton buffering and removal, this causes exercise‐induced acidosis (Robergs et al., 2004). Furthermore, glycolysis generates pyruvate molecules, which are catalyzed to lactate. The production of lactate consumes protons and works as a buffer against exercise‐induced acidosis (Robergs et al., 2004).

Because our results showed elevated lactate levels after each resistance exercise suggesting anaerobic ATP production and the formation of exercise‐induced acidosis, we wanted to investigate if lactate or possible acidosis mediated the observed changes in cellular respiration of PBMCs to help explain our main results (Figures 2 and 3). Although we did not measure the actual pH change of blood during the experimental resistance exercises, we aimed to replicate the exercise‐induced environment with lactic acid concentration ex vivo. However, our findings indicated that neither lactate nor the expected decrease in pH had a significant impact on the cellular respiration of PBMCs (Figure 5). Other factors related to peripheral muscle fatigue, such as production of reactive oxygen species (Finsterer, 2012), and the switch from oxidative phosphorylation to glycolysis could have a role in bioenergetics of PBMCs and should be investigated.

### 4.4 Exercise‐induced stress

Acute resistance exercise causes a temporary stress condition, which activates the hypothalamic–pituitary–adrenal (HPA) axis eventually leading to elevated cortisol levels (Becker et al., 2021; Izquierdo et al., 2009). Cortisol and catecholamines bind to special receptors on the surface of immune cells in the organ reservoirs and vascular walls, causing reduction in adhesion molecule expression, which eventually leads to leukocytosis (Gleeson et al., 2013). This phenomenon is seen in our results as the amount of white blood cells measured from whole blood samples increased significantly after each resistance exercise (5 min post‐exercise response) (Table 5). Cortisol and catecholamines have a major impact on the migration of leukocytes (Gleeson et al., 2013) and might also affect other levels, such as the metabolic level. It has been observed that the greatest cortisol response to acute resistance exercise is detected during high‐volume sessions with moderate or high intensity (Kraemer & Ratamess, 2005). Being the exercise with the highest workload, E + C caused the greatest elevation in white blood cell amount and this was also statistically significant when compared to E and C (Table 5), which indicates a more stressful exercise. Although we did not measure the change in cortisol levels, our white blood cell counts (Table 5) and lactate levels (Figure 4c,d) suggest that the expected metabolic and systemic stress from acute resistance exercise influence bioenergetics of PBMCs, and the intensity and volume of resistance exercise has a major role in this process.

### 4.5 PBMC composition

From PBMCs, lymphocytes form the main cell group, having frequency of 70%–90% while monocytes accounts for 10%–20% and dendritic cells 1%–2% of PBMCs (Kleiveland, 2015). Under basal conditions, it has been shown that lymphocytes have an oxidative nature whereas monocytes utilize both oxidative phosphorylation and anaerobic glycolysis (Chacko et al., 2013; Kramer et al., 2014). Moreover, lymphocytes have smaller reserve capacity than monocytes, indicating different metabolic programming in ATP production (Kramer et al., 2014). Our results showed that the number of lymphocytes was elevated after each resistance exercise but E + C elevated these numbers significantly more than C (Table 5). This is also seen as a higher percentage change after E + C. However, as described earlier, E + C in particular decreased the cellular respiration of PBMCs and C had a similar effect, despite not reaching statistical significance. As lymphocytes have oxidative metabolism, their increase may not explain the decreased cellular respiration of PBMCs. However, we did not measure the portion of anaerobic glycolysis, therefore our results do not explain the possible role of metabolic switching in the observed changes due to resistance exercises. Also, the pool of lymphocytes probably consisted of already circulating lymphocytes and those coming from the organ reservoirs. This may have some effect on the activation status of these cells and their final destination into tissues needing immune surveillance after exercise.

Monocytes increased significantly after C and E but there was no difference in response between resistance exercises (Table 5). Moreover, the percentage of monocytes remain approximately the same after all resistance exercises. As there were no significant changes in monocyte counts between exercises, our results suggest that monocytes alone did not cause the observed changes in cellular respiration.

## 5 CONCLUSIONS

Acute resistance exercise in the form of eccentric‐only versus concentric‐only and eccentric‐concentric contractions have distinct effects on PBMCs that are observed in the respiration profile of these cells. As expected, combined eccentric‐concentric exercise was the most stressful resistance exercise based on increased blood lactate and decreased maximum isometric force, indicating the greatest muscle fatigue. Eccentric‐concentric resistance exercise led to significant reductions in PBMC respiration, and a similar pattern of PBMC respiration and fatigue/lactate response, although smaller in magnitude, was observed from concentric‐only exercise. In comparison, eccentric‐only exercise led to the smallest change in both lactate and isometric force and moreover, had no effect on acute respiration states. This divergent response pattern led to statistically significant differences between eccentric‐only and the other two exercise modes immediately after exercise. The effect of exercise‐induced anaerobic metabolism was further studied via lactate treatment and change in pH ex vivo, but these did not affect cellular respiration of PBMCs. Finally, combined eccentric‐concentric exercise increased the number of lymphocytes and slightly changed the proportion, but this response did not help to explain the observed changes in cellular respiration. Future studies should concentrate on the mechanism behind the observed changes in cellular respiration of PBMCs. According to our results, neither (1) exercise‐induced inflammation nor (2) anaerobic metabolites were contributing factors to the acute respiration responses.

## AUTHOR CONTRIBUTIONS

Experimental design: M.L., S.W., E.I.L., M.K. and I.K. Investigation and data collection: E.I.L, M.K., I.K., E.L. and A.K. Writing and manuscript preparation: E.I.L, M.K., A.K., H.K., S.W. and M.L. Review and editing of the manuscript: S.W., M.L., E.I.L. and H.K. Funding acquisition: H.K. All authors have read and agreed the final version of the manuscript.

## CONFLICT OF INTEREST

No conflicts of interest are declared by the authors.
